# Service Providers’ Perspectives of an Integrated Community Mental Health Service in the UK

**DOI:** 10.1007/s10597-024-01352-w

**Published:** 2024-09-13

**Authors:** Taru Silvonen

**Affiliations:** 1 Population Health Sciences, University of Bristol, Oakfield House, Oakfield Grove, Bristol, BS8 2BN UK; 2 Present Address: Faculty of Science & Engineering; School of Electrical, Electronic and Mechanical Engineering, Queens Building; University Walk, Bristol, BS8 1TR UK

## Abstract

This qualitative service evaluation aims to provide in-depth insights of service providers’ perspectives of a new integrated community mental health service piloted in one NHS Integrated Care Board locality in South West England, UK, considering to what extent the service is meeting the mental health support needs of adults who are in between primary and secondary care services. In total, 21 semi-structured remote interviews were carried out in June-August 2023 with service providers and lived experience representatives. The evaluation was carried out through a researcher in residence -placement. Qualitative analysis drew on framework and thematic analysis, which was completed using Nvivo 20. Thematic analysis drew on a framework of context, mechanisms and intended or unintended consequences. These showed that service providers’ positive expectations of the service were undermined by insufficient integration, which was evident from the imbalances in information flow and presence of pre-existing provider specific practices. The evaluation found several improvement opportunities: a need for deepening integration beyond the initial service development phase; aligning working practices with service delivery aims and ensuring that new services are not rolled out prematurely before service delivery practices have been fully developed.

## Introduction

Mental health support services provided through the NHS have been under increasing pressure since austerity measures in the UK (Cummins, 2018; Tallack et al., 2020). Simultaneously, several government strategies have started to shift care from the NHS to the community (Ebrahimoghli et al., 2023), making community mental health teams a key part of mental health service delivery (NHS England and NHS Improvement and the National Collaborating Central for Mental Health, 2019). However, community mental health services have been criticized for having fragmented approaches to service delivery – primarily due to the high number of voluntary and third sector provision that is offering different services within a broad remit – that strategies focusing on integration of care have sought to respond to (Mechanic, 1997; NHS England and NHS Improvement and the National Collaborating Central for Mental Health, 2019).

Integrated service models bring together “primary and multi-disciplinary psychiatric care through different service units” (Chiang et al., 2020, p. 729). Common features of integrated care models often include “multidisciplinary meetings, care coordination”, “joint treatment”, “and person-centred care” (Coates et al., 2020, p. 38). Integrating mental health services bring together the benefits of services such as social prescribing which is designed to provide non-clinical mental health support (Ebrahimoghli et al., 2023; Hazeldine et al., 2021). As such, integrated services acknowledge the variety of factors affecting mental health, aiming to provide care in a holistic manner (Cummins, 2018; Stansfield et al., 2021). There is some indication that integrated community mental health services (ICMHS) are able to provide support in terms of access to services but the clinical effectiveness of ICMHS is somewhat lacking (Chiang et al., 2020).

This qualitative service evaluation aims to provide in-depth insights based on service providers’ experiences of the new integrated mental health service piloted in one NHS Integrated Care Board (ICB) locality in South West England, considering to what extent the service is meeting the mental health support needs of adults who are in between primary and secondary care services. The evaluation focuses on a service that is being piloted and is still in development, which is why this is an implementation-focused formative evaluation focusing on the operationalization of the new service (Elwy et al., 2020). The results of this evaluation outline challenges and enablers relevant in the development of integrated community mental health services more broadly.

### Background

Weston, Worle and Villages (WWV) is one of six Bristol, North Somerset and South Gloucester (BNSSG) NHS Integrated Care Board (ICB) localities (BNSSG ICB, 2023). Each locality is expected to deliver an Integrated Delivery Plan that sets out their respective approaches to integrated community mental health (BNSSG ICB, 2022). The integrated mental health team MINT Hub (Hub) was set up as a pilot in WWV, which is the first BNSSG locality to roll-out their new integrated community mental health service model. The integrated service brings together primary care services including Primary Care Liaison Service (PCLS), mental health services such as a psychologist, clinical associates in psychology (CAPs), occupational therapy, adult social care, Talking Therapies, recovery navigators (Second Step), drug and alcohol rehabilitation as well as social prescribing through voluntary and community sector services (VCSS). This combination of services was expected to address the service needs of those who do not meet the threshold for secondary care services but who may not receive the support they need from primary care services. The integrated model was developed to provide proactive and preventive mental health support through the provision of earlier targeted services and streamlined interactions between those using services as well as service providers across different organisations (BNSSG ICB, 2023). Overall, the integration aimed to improve service users’ experiences of care, reduce demand for services and improve cost-effectiveness of services (ARC West, 2020). Partnership working involved a shared caseload for which the clinical risk would also be shared; initially making first contact within 24 h; a 4 week treatment response time; and focus on the ‘team around me’ approach to work with patients through co-produced care plans (One Weston, 2021). The first referral to the service was received in October 2022 as the service gradually opened to referrals initially from the PCLS and GPs across the locality.

A researcher-in-residence placement was set up to fund the role of an independent researcher to evaluate the new integrated service during the first year of service provision. Researcher-in-residence placements – or embedded researcher placements (Woodall et al., 2024) – are seen as an opportunity for co-production of knowledge through researcher-practitioner partnerships as a researcher is embedded into a healthcare organisation whilst applying research skills to analyse, for example, care services in a specific local context (Vindrola-Padros et al., 2019). While there is a strong evaluation culture within NHS England and the BNSSG ICB has its own research and evaluation team, bringing in an independent evaluator to carry out the service evaluation often reduces the risk of deliberate as well as unintentional bias (Mayne, 2012).

## Methods

Semi-structured one-to-one remote interviews were the main data collection method for this qualitative evaluation. Ethical approval was not sought as per the guidance regarding service evaluations (ARC West, 2020; Eden & Lowndes, 2013). As recommended by the NHS BNSSG ICB, a peer review of the evaluation protocol was carried out by members of the BNSSG ICB Clinical Effectiveness Team resulting in a supportive letter (Ref: CE23.005 CMHP) being issued to the researcher 23/05/2023. The researcher was also contracted to carry out the work through an honorary contract and an NHS research passport (NHS Health Research Authority, 2019) process which combined provided the researcher with the required IT access. Following ethical practice, participants were provided with participant information via email before they were asked to complete an online consent form via MS Forms. Participant recruitment was supported by an introductory email that was sent on behalf of the researcher to a staff mailing list by the Hub manager, stating that they may be contacted directly by the researcher. The introductory email was followed by individually sent emails that were sent out on a rolling basis, gradually inviting staff from the different participant categories (see Table 1) to read the participant information sheet and consent form. Once potential participants shared their availability, the researcher followed this up with a calendar invite including a video calling link.

### Data Collection and Participant Characteristics

Interviews were carried out in June-August 2023 via video calling using MS Teams with only one interview taking place over the phone. All interviews were recorded and professionally transcribed. No demographics information was collected from the participants to protect their anonymity. The participants included core Hub staff who were based at the Hub headquarters and staff from partner organisations (see Table 1 below): psychologists; CAPs; occupational therapists; service managers or clinical leads; and other service providers such as recovery navigators and a social worker. As the service development included input from lived experience representatives (LERs), LERs were also interviewed as part of the evaluation. In total, 35 professionals, including staff at three GPs, were invited to take part in the interviews. Those who declined explained that this was due to time pressures or lack of first-hand experience of the Hub.

The partner services that were represented in the evaluation included a local authority, a local Citizens Advice Bureau, a rehabilitation service and voluntary and community sector organisations.

**Table 1:** Number of participants across role categories

Role	N participants
Hub staff	7
Partner staff	11
LERs	3
TOTAL	21

Due to challenges in engaging service users in the evaluation through interviews, this paper focuses only on the service-provider and lived experience representatives perspectives.

### Data Analysis

Interview transcripts were analysed using Nvivo software following a coding framework that was adapted from realist evaluation studies (Crampton et al., 2019; Lemire et al., 2020). The principles of framework analysis (Gale et al., 2013) were followed, so that while analysis focused on the key topics identified by the ICB, further codes were formed based on constant comparison of the meanings conveyed by the participants. In this sense, the analysis was inductive and data-driven. Drawing on an analytical framework based on realist evaluation, the broad analytical themes were: contexts, mechanisms and outcomes. Each theme was expanded to 2–4 sub-themes and furthermore to 5–8 codes. While the analysis framework was discussed with an independent researcher, it was not possible to review data coding in the same manner due to resourcing issues.

### Limitations to Data Collection

The evaluation placement involved two days of researcher time per week during 03/04/2023-13/10/2023. At the beginning of the evaluation, the service was receiving direct referrals from the Primary Care Liaison Service and certain GPs across the locality. Referrals from GPs were rolled out gradually during the evaluation but when interviews were completed in August, the roll-out had not yet completed.

## Results

The results are discussed focusing on the most prominent sub-themes listed in Table 2.The less prominent sub-themes that are not discussed here due to relatively limited evidence included: (1) context: ‘wider welfare system’; (2) programme mechanisms: ‘unclear functions’ and ‘future reflections’; (3) unintended consequences: ‘broader institutional setting’ and ‘overlap with services’.

**Table 2:** Sub-themes discussed in results

Theme	Sub-theme
Context	Institutional
	Interpersonal relationships
Programme mechanisms	New ways of working
	Partner services
Intended consequences	Responsive support
Unintended consequences	Limited remit

### Context: Institutional

When discussing their roles within the Hub service and their overall views of the service, the participants raised several aspects that related to their previous experiences of working in health care and which reflected the organisations they are primarily employed by. The aspects focused on experiences of patient care, waiting times and working practices, including operational procedures. These contexts often supported the rationale for the service model:

“I’ve worked previously in secondary services, and I’ve worked in primary care liaison. So I can see the people that are coming through to secondary services that may not necessarily need to but there was nothing else there, but also the amount of people we’ve discharged back to the GPs after a primary care liaison assessment, and not offered them some signposting in the hope that they will do that themselves…the model itself, I think it’s been well thought out and it feels like, on the whole, that there’s been a good understanding of what the community of Weston needs from a mental health perspective” (P21).

Some highlighted initial barriers to integrated working which stemmed from the specific working practices within individual services that had not been amalgamated into broader shared practices across the Hub partnership. One example of this was different ways of measuring performance:

“We have our own measuring tool outside of the Hub, but it will be eventually integrated to use in the same one as the Hub. But yeah, overall the clients that we’ve worked with that have completed the 6–8 weeks intervention have identified and had a 90% increase in mental health and wellbeing” (P14).

### Context: Interpersonal Relationships

Interpersonal relationships and the interactions amongst especially the staff who were physically based at the Hub supported peer learning. Participants felt it was beneficial that staff from different backgrounds were able to contribute something to the integrated service and this formed a basis for collaborative working.

“And everyone has something different to bring; different opinions, different experiences, different learning to share, and that will help with integration as well because, like I said, it’s not about this person has been to uni [university] and got a degree and someone in our team hasn’t, that shouldn’t mean that they don’t have a voice or anything like that” (P10).

Forging new professional relationships across the different service providers involved in the Hub was seen as a positive way of working. Forming these connections was a necessary and welcome step towards collaborative working:

“We did have a team lunch as well where we all got together with the integrated mental health hub and our peer health social prescribing team and also our virtual hub that’s just being set up in North Somerset just to learn about, you know, everybody, and bring everybody together and learn about what each other does so we could work a bit closer together” (P16).

### Programme Mechanisms: New Ways of Working

Working as part of the Hub provided opportunities for professional development and new ways of working which for some brought greater autonomy to their approach to treatment:

“Being in the Hub has allowed me to be a bit more flexible with my caseload… my cases would be screened previously, they would be triaged, and they would be allocated. I now am needing to use my initiative as to whether it’s [a referral] appropriate to a social care service or not” (P1).

The new ways of working, especially the commitment to contact new patients within a short timeframe, were also seen as something that could potentially be beneficial for patients:

“But if you can be seen, I think they had to increase it to 48 [hours] now. So just to be talked to within that short time. And then for them, the people, to get that support frequently and regularly after that” (P13).

However, some concerns were also raised about the commitment to new ways of working that were incorporated in the standard operating procedure (SOP), and whether these, including the holistic approach to patients’ needs could be met:

“Things are much more about the person as a whole. I think that is really problematic, because I think it looks beautiful in the document and everyone loves to talk about it… But some of the people we’re talking about, it’s very very hard to work and engage with them, and imagining them on an allotment project is not easy…I think there’s a danger that people’s problems are sort of slightly downplayed in some of this work” (P2).

These concerns related to whether patients were able to say what kind of treatment they would like as well as whether something like group activities would be suitable for people with potentially long-term or complex needs.

### Programme Mechanisms: Partner Services

Participants were positive about the aim to bring together services across different sectors. However, the integration of different services was still seen to be in development:

“I feel there potentially needs to be a bit more involvement from voluntary sector… you get other more specialist services like drug and alcohol services, social prescribing, for example” (P10).

Partner organisations themselves were keen to be more involved, which suggests that the integration of different services was still in development despite the service being up and running:

“We’re an outside agency who’s invited to meetings and we’re there to give input and advice… if you’re really looking at it being as integrated into a mental health hub then maybe, you know, we need to be a bit more integrated” (P15).

Some concerns were raised about whether the different partner organisations shared clinical practice and whether their working practices aligned sufficiently to provide cohesive support. While the Hub operates based on its own SOP, there were differences in the practice of clinical and non-clinical partners:

“Yes, I suppose it’s what’s the shared governance that’s going on here between all the services. Do we all know that we’re offering treatments that are NICE compliant and have the right oversights and stuff? At the moment we don’t have anything” (P4).

### Intended Consequences: Responsive Support

Central to the Hub service was the aim to contact new patients within 48 h, and to plan support around patients’ needs through an initial support conversation between the patient and a service provider. The service providers felt that providing responsive support was an improvement.

Participants felt that one of the key benefits of the Hub service was the responsive support it offered through a combination of active signposting and the aim of soft referrals which should make the transition between different services easier for the patient:

“Going back to pre-Hub days and the way clients potentially could be bounced around services until they got to the right service. I think the Hub is… it’s a massive improvement in the way of getting clients to the right service in the shortest possible time to meet their support needs” (P14).

Collaboration with partner organisations was seen to support finding a suitable service for patients. A joined up way of working could potentially support finding the best fitting service to meet patients’ need:

“If I feel I can’t support somebody, maybe my role isn’t appropriate for a referral or something, it’s nice that it’s kind of easy to just signpost them to the services that are linked in with us” (P17).

### Unintended Consequences: Limited Remit

The main unintended consequence, or outcome of the service that was not expected to occur, related to the limited remit of the Hub service, despite the aim to address the existing service gap between primary and secondary care. This was linked to the limited capacity of the Hub in terms of staffing resources and the unexpected demand for the Hub service:

“So my only fear is that the more the Hub is utilised, there isn’t necessarily additional capacity to meet the demand. So, then you run higher risk of higher waiting lists and things. And then even if you’re trying your best to meet that need early, you know, you can accept it, but you can’t see them [the patient] for another six months, for example” (P10).

Limited capacity also extended to the Hub’s inability to deal with complex patients due to not offering a specialist service that was fit to support patients with complex needs such as long-term trauma:

“If someone’s too complex for primary, but not complex enough for secondary, that’s kind of where they fall for us… I’d say like almost everyone who’s been referred to us has some type of trauma, but we are not commissioned to treat that trauma or, you know, we don’t have the staff who can help with that trauma” (P17).

Further integration with mental health services was suggested to bridge the existing service gaps that remained despite the introduction of the Hub service:

“I think we need to integrate more with our mental health service colleagues… I think we need to integrate more with the recovery team, the crisis team and things like that” (P21).

## Discussion

This qualitative service evaluation focused on understanding service providers (SPs) views and experiences of the Hub service regarding the extent to which the Hub service was able to meet the mental health needs of adults who are in-between primary and secondary care services.

The interviews with SPs, including LERs, showed that the Hub was able to bring together SPs across many professional backgrounds. Peer learning was supported by regular interaction especially for those professionals who had a physical presence at the Hub. Sharing expertise supported professional development. However, the partner services still had their own working practices which they had to follow primarily, suggesting incomplete integration of practices.

The results show that while SPs saw a need for a new as well as an integrated service, the level of integration within the service remained underdeveloped. This was evident from the two prominent programme mechanism subthemes: new ways of working and partner service. The caveats related to the varying level of involvement of different partners in the service, in addition to disconnected working guidelines and clinical practices. Especially more involvement from the VCSS was felt to be needed, whereas partners from the VCSS were keen to become more involved. Complex systems which involve several stakeholders regardless of the sector they work in are prone to issues relating to power dynamics and competing worldviews (Barnes et al., 2003). In the Hub, finding common ground was initially supported via informal meetings but these lost prevalence as the service moved from the early development phase to gradually rolled out service provision. While the starting point for integration was established, this work was not continued as the service became busier. It is unclear why the initial conversations that appeared to support integration were not continued throughout service delivery.

In terms of the intended consequences of the Hub service, the SPs felt that the service was needed and had the potential to meet patients’ needs at least partially, suggesting that there is scope for the Hub to address the identified gap in service provision. Central to this service was providing care in a responsive manner, which reflected the SOP of the Hub service.

However, the unintended consequences of the Hub service highlighted the limited remit of the service. Whilst the Hub was developed to respond to a gap in service provision, the limitations within the new service meant creating a new, more niche gap. Especially lack of trauma support and support for patients with complex needs were highlighted by the SPs. Similarly, evaluation focusing on social prescribing only has emphasized the difficulty of capturing to what extent social prescribing interventions are able to meet complex needs (Polley et al., 2017). Overall, the barriers to service delivery outlined in this service evaluation reflect those discovered in previous research on integrated services. Integrated community mental health teams have been shown to struggle with gaps in how “team structures and characteristics to quality care” are understood (Wilberforce et al., 2011, p. 221). As the Hub SPs commented, integration takes time and continuous effort. It was evident that efforts to integrate different professionals into the service were concentrated to the initiation of the Hub, which was not sufficient to form collaborative working practices in the long run. The importance of continuous activities to improve the quality of implementation has been emphasized especially in community mental health services (Carlson et al., 2012). Managers and clinical leads can play a key role in liaising with different SPs to clarify roles across multi-disciplinary services (McCrae et al., 2008). This can support mitigating any arising concerns about differing standards of practice, enabling services to provide “the right service at the right time”, which is often seen as one of the strengths of integrated community mental health services (Chiang et al., 2020, p.737).

The learnings from the evaluation of this local service in South West England are applicable in the broader context of mental health service provision due to the practical aspects relating to the integration process. While the integration of services is a welcome response to challenges in adult mental health service provision, bringing together partners with differing practices will require deep collaboration and commitment to constant development beyond the initiation of a new service. This will inevitably take up staff time in addition to the daily responsibilities, emphasising the need for allocating sufficient resources to meet both the needs of patients and service development needs. Finding the time to deepen the integration of services can be challenging, considering that in 2019, 44% of mental health service providers in England felt they were unable to meet inpatient service demand, with this rising to 58% in community mental health services (McDaid & Park, 2022, p. 43). The co-location of community mental health services has been shown to have many benefits from improving access for patients (Burrows et al., 2011) to reducing stigma related to mental health support by creating safe and friendly environments (Baskin et al., 2023). While a coordinated approach across different mental health service providers – including voluntary and third sector services – is clearly needed to improve preventative care that cuts across different risk factors (Duncan et al., 2021; McDaid & Park, 2022), the continuous effort required to develop genuinely shared practices should not be underestimated when integrating existing services.

The strengths of this study lie in the qualitative approach to data collection and analysis which resulted in a detailed picture of SPs experiences across a variety of services involved in delivering the service. Having an independent researcher carry out the evaluation mitigated risk of bias and allowed SPs to talk freely during the interviews.

Limitations of the study include the evaluation being carried out whilst the service was still in development which also affected the limited integration of the researcher-in-residence placement. The gradually increasing number of referrals resulted in increasing pressure on staff which is why the results of the evaluation should be considered in light of the phase of service development. Furthermore, this meant that the integration of the researcher-in-residence placement was limited by staff members’ fluctuating capacity to support the evaluation work. As stated in the methods section, this paper also does not include service users’ perspectives of the service due to data quality issues as a very small number of people agreed to be interviewed or consented to having the interview recorded. The challenges to involve service users in the evaluation could have been mitigated by more practitioner-researcher interaction at the beginning of the placement (see, e.g. Vindrola-Padros et al., 2019, p. 71) to support the recruitment of service users.

## Conclusion

While this service evaluation highlighted that there is a need for mental health services that address the gap between primary and secondary care provision, SPs identified some challenges in the service developed to address this gap. SPs recognised the need for a new service and mostly welcomed opportunities to work in an integrated way. However, incomplete integration in terms of involvement of partners and shared working practices such as clinical guidance limited service provision. These challenges should be addressed to ensure the service can better meet the support needs of adults who fall in between primary and secondary care services.

In the development of integrated services more broadly, it is recommended that standard operating practices are streamlined across different SPs and processes put in place to review practices regularly to support operating an integrated service. It is also recommended that the development of new integrated services includes an implementation plan for continuous integration improvement to: (i) address emerging imbalances in SP partnerships; and (ii) maintain information flow between SPs to facilitate further peer learning.

## Data Availability

Due to this being a service evaluation, data are not available for research purposes.
